# Dye Sorption from Mixtures on Chitosan Sorbents

**DOI:** 10.3390/molecules29153602

**Published:** 2024-07-30

**Authors:** Urszula Filipkowska, Tomasz Jóźwiak

**Affiliations:** Department of Environmental Engineering, University of Warmia and Mazury in Olsztyn, Warszawska St. 117a, 10-957 Olsztyn, Poland; tomasz.jozwiak@uwm.edu.pl

**Keywords:** reactive dyes, chitin, chitosan, mixtures of dyes

## Abstract

This article presents studies on the sorption of the anionic dyes Reactive Black 5 (RB5) and Reactive Yellow 84 (RY84) from solutions of single dyes and from dye mixtures onto three chitosan sorbents–chitin, chitosan DD75% and chitosan DD95%. In this work, the influence of pH on sorption efficiency, the sorption equilibrium time for the tested anionic dyes and the sorption capacity in relation to the individual dyes and their mixtures were determined. It has been found that the sorption process for both dyes was most effective at pH 3 for chitin and chitosan DD75 and at pH 4 for chitosan DD95%. The obtained results were described by the double Langmuir equation (Langmuir 2). The obtained constants made it possible to determine the affinity of the tested dyes for the three sorbents and the sorption capacity of the sorbents. For RB5, the highest sorption capacity for chitosan DD95% was achieved with sorption from a single solution–of 742 mg/g DM and with sorption from mixed dyes–of 528 mg/g DM. For RY84, the highest efficiency was also achieved for chitosan DD95% and was 760 mg/g DM for a single dye solution and 437 mg/g DM for a mixture of dyes.

## 1. Introduction

Dyes are defined as chemical compounds of natural or synthetic origin that selectively absorb electromagnetic radiation in the wavelength range of 400–700 nm. These compounds have the ability to give colour to various materials. They are used in the textile, tanning, textile, paper, pharmaceutical, plastics and cosmetics industries [1,2]. The properties of synthetic dyes are determined by their chemical structure. Dyes have chromophore groups that selectively absorb electromagnetic radiation—they cause the production of colour and auxochrome groups that are responsible for the affinity to coloured materials, improve the durability of the colour and give the dyes properties such as solubility in water or organic and mineral solvents [3]. Anionic dyes are compounds that dissociate in aqueous solutions and form a coloured anion. They are characterised by an intensive and permanent colour and very good solubility in water [4]. Among the anionic dyes, there are reactive dyes in the form of organic salts, bases and acids. They are characterised by their ability to form a permanent chemical bond with the dyed material thanks to the presence of active groups in the molecule, e.g., vinyl sulphonic acid. They are used for dyeing cellulose fibres, wool and silk [5]. 

Among the methods for decolourising coloured wastewater there are the following: biological and physicochemical [6]. Biological methods use reactors with activated sludge or biofilm beds to treat coloured wastewater. The effectiveness of this group of methods depends on the concentration of dyes and the residence time in the system. Biological methods are characterised by low efficiency, which is due to the low susceptibility of the dyes to biodegradation. Biological treatment processes for coloured wastewater are generally lengthy [7]. The toxic effect of some dyes on microorganisms can also contribute to low treatment efficiency.

Physicochemical methods of wastewater decolourisation include coagulation, whose disadvantage is a large amount of sludge formation [8,9]; electrocoagulation, whose main disadvantage is high electricity consumption [10]; membrane methods, whose disadvantage is the high price of membranes, the need to ensure high pressure during the process and high water losses [11]; ozonation, whose disadvantage is the possibility of formation of organic compounds with toxic and carcinogenic effects [12]; and sorption, whose effectiveness depends mainly on the appropriate choice of sorbent. Sorbents can be waste products [9,13] from other industries that can be used in sorption processes to remove contaminants such as dyes, heavy metals or nutrients before final disposal [13,14]. These sorbents include chitosan sorbents. Chitin is a polysaccharide consisting of N-acetylglucosamine and forms a hard biomaterial that occurs everywhere in nature. Chitin is the second most abundant polymer after cellulose. Chitin and cellulose have a similar structural composition, with the exception that the hydroxyl groups attached to the carbon atom in chitin are replaced by an acetamide group. This allows for increased hydrogen bonding between neighbouring polymers. As a result, chitin is characterised by higher strength compared to cellulose [15].

Chitin forms the skeletons of crabs, lobsters and shrimps. It is also found in insects (e.g., ants or beetles) and cephalopods (e.g., squid and octopuses) or in the cell walls of fungi. On an industrial scale, however, chitin is obtained as a waste product during the processing of marine invertebrates such as crabs, shrimps, lobsters and krill [15].

Chitin is characterized by properties such as bioactivity, biodegradability and membrane- and fibre-forming. It also has sorption, chelating and antibacterial properties. Thanks to these properties, chitin has been widely used in medicine, pharmacy, cosmetics, textile and paper, and food industries [15,16,17].

Chitosan is a polysaccharide, a derivative of chitin, which is formed by partial deacetylation. This process involves the exchange of acetamide groups (which form the structure of chitin) for amino groups. The sorption capacity of chitosan changes with the degree of deacetylation (DD) and the number of amino groups.

Due to the presence of primary amino groups in the structure of chitosan, it adsorbs metal ions and many classes of dyes very well. For example, 1 g of properly prepared chitosan can bind 2 g of anionic dyes [18].

Chitosan is used as a sorbent for the decolourisation of coloured wastewater due to its properties such as non-toxicity, biocompatibility, biodegradability, antibacterial activity and high sorption capacity. Chitosan is also used in medicine, the textile and paper industry as well as in the food industry [19].

The form of the chitosan (flakes vs. hydrogel granules) and the degree of deacetylation influence its sorption capacity. Hydrogel granules with a loose structure and easy access to the sorption centres showed higher sorption capacities compared to flakes [1,20]. 

In post-dye wastewater, dyes are found individually or in two- and multi-component mixtures [20,21,22,23,24,25]. Compared to single dyes, the adsorption of mixtures can be complex, both due to the potential for interactions between the dyes in solution and their ability to compete for active sites on the adsorbent surface. In addition, the adsorption of each individual dye can alter the surface charge of the adsorbent and consequently cause a decrease or increase in the binding efficiency of other dyes present in the mixture.

In this study, the adsorption efficiency of a mixture of reactive dyes Reactive Black 5 (RB5) and Reactive Yellow 84 (RY84), popular in the textile industry, by chitin and chitosan with different degrees of deacetylation was investigated.

## 2. Results and Discussion

### 2.1. FTIR Analysis

**Figure 1:**
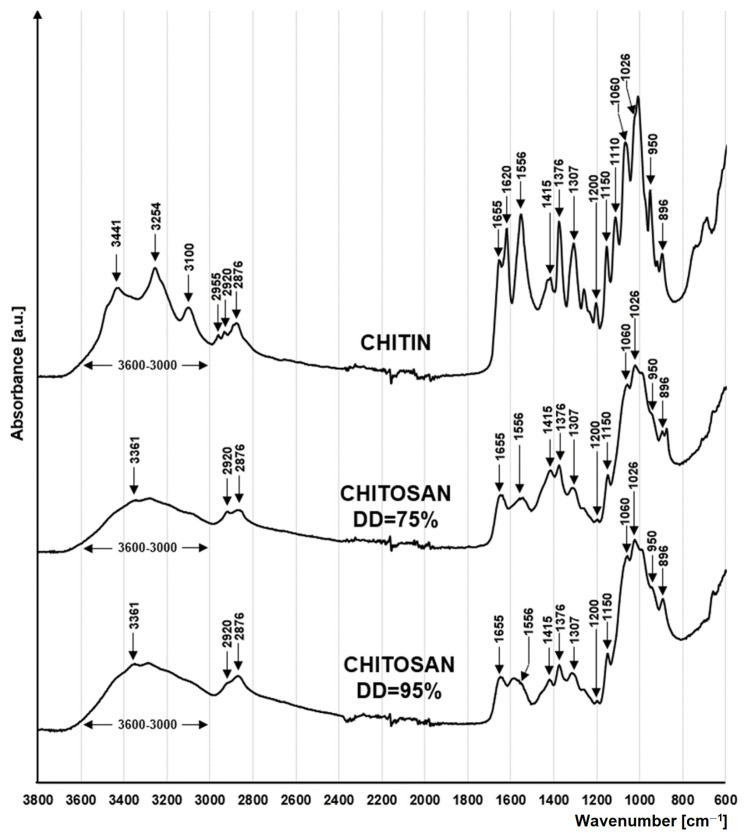
FTIR spectra for chitin, chitosan DD = 75% and chitosan DD = 95%.

The FTIR spectra of the tested materials are typical for polysaccharides (Figure 1). The peaks at 1060 cm^−1^ and 1026 cm^−1^ indicate the stretching of the C-O bond on the C3 and C6 carbons of the saccharide rings [26]. The peaks at 1150 cm^−1^ and 1200 cm^−1^ are attributed to the asymmetric and symmetric stretching of the glycosidic C-O-C bond between the pyranose rings [27]. In contrast, the peaks at 1110 cm^−1^, 950 cm^−1^ and 896 cm^−1^ are attributed to the stretching of the skeletons of the saccharide rings [27,28]. Peaks at 2920 cm^−1^ and 2876 cm^−1^ indicate symmetric and asymmetric stretching of the C-H bond in the side chains of chitin and chitosan (-CH_2_-OH) [27,29] and deformation of CH_2_ within these chains [30]. A wide absorption band of 3500–3000 cm^−1^ is attributed to the stretching of the O-H bond of hydroxyl functional groups [26].

Peaks associated with the presence of the acetamide functional group (-NHCOCH_3_) are characteristic of the chitin spectrum. These include peaks at 2955 cm^−1^ and 1376 cm^−1^ indicating the presence of the -CH_3_ group [31], peaks at 3254 cm^−1^, 3100 cm^−1^ corresponding to the N-H bond of the amide, as well as peaks at 1655 cm^−1^ and 1620 cm^−1^ indicating the presence of a C=O bond [32], as well as a peak at 1307 cm^−1^ indicating the stretching of the C-H bond [33]. Peaks associated with the presence of acetamide groups are much lower in the chitosan spectra or are obscured by neighbouring peaks (Figure 1). The effect obtained is related to the high degree of deacetylation of the tested chitosan (DD = 75%/DD = 90%).

A characteristic feature of chitosan is the primary amino groups that are formed as a result of deacetylation. The presence of -NH_2_ groups in the material is evidenced by the peak at 3361 cm^−1^ [34], which is best seen in chitosan with DD = 95%.

### 2.2. Effect of pH on Sorption Efficiency

**Figure 2:**
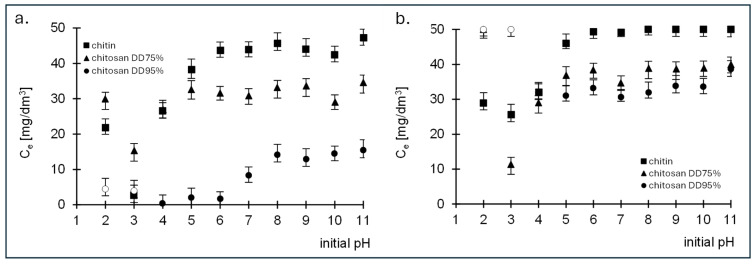
Effect of pH on the effectiveness of sorption of (**a**) RB5 and (**b**) RY84 onto chitin, chitosan DD75% and chitosan DD95% (sorbent dose 1 g/dm^3^).

The study of the optimal sorption of the dyes RB5 and RY84 by chitin, chitosan DD75 and chitosan DD95% was carried out in a pH range from 2 to 11. The duration of sorption was 180 min. The initial concentration of the two dyes was 50 mg/dm^3^. The results are shown in Figure 2.

The RB5 dye was removed most efficiently at pH 3 with both chitin and chitosan DD75 (Figure 2a). In contrast, chitosan DD95% was found to have the lowest final concentration at pH 4 (Figure 2a). Based on the results obtained, it is clear that the amount of RB5 dye removed decreases with increasing pH, regardless of the sorbent used. The high efficiency of RB5 sorption on the tested sorbents at low pH is the result of a positive charge on the surface of the sorbents. The protonated functional groups of the sorbents electrostatically attract the anionic dye RB5, which increases the sorption efficiency. At high pH values, the surface of the sorbent acquires a negative charge, whereby the anionic dye is repelled and can only be absorbed with difficulty. In case of the second dye tested, RY84, the highest sorption was also observed at pH 3 for chitin and chitosan DD75 and at pH 4 for chitosan DD95% (Figure 2b).

Based on the adsorption results obtained, a pH value of 3 for chitin and chitosan DD75 and a pH value of 4 for chitosan DD95% were assumed for further studies.

Both decreasing the pH to 2.0 and increasing it had a negative effect on the amount of bound dye, regardless of the type of adsorbent.

For chitosan adsorbents, a strongly acidic environment (pH 2) proved to be unfavourable and led to a deterioration in the mechanical properties of the adsorbent, resulting in its destruction.

The binding efficiency of the dyes was related to the protonation of the amino groups of the chitosan according to the following reaction:

R − NH_2_ + H^+^ ↔ R − NH_3_^+^

At the same time, one molecule of the reactive dye dissociates:

D − SO_3_Na → D − SO_3_^−^ Na^+^

The adsorption process therefore took place through an electrostatic interaction between the two molecules:

R − NH_3_^+^ and D − SO_3_^−^

R − NH_3_^+^ + D − SO_3_^−^ ↔ R − NH_3_^+^ − SO_3_^−^ − D

Increasing the pH of the solution reduced the electrostatic interaction due to deprotonation of the amino groups. However, chitosan continued to adsorb the dye molecules at pH 6–11, but to a lesser extent. This can be explained by a combination of other interactions such as van der Waals forces and hydrogen bonding. In a strongly alkaline reaction, the hydroxyl groups of chitosan were deprotonated according to the following reaction:

−CH_2_OH + OH^−^ ↔ CH_2_O^−^ + H_2_O. 

The binding mechanism of anionic dyes to chitin follows the same reactions. The only difference is that, due to its high degree of acetylation, chitin mainly contains −CO−NH− amide groups, which is not as easily protonated in acidic solutions as the amino groups of chitosan.

Based on the results from the first phase of the study, it can be concluded that the pH value of the aqueous solutions of dyes has a significant influence on sorption efficiency. For both dyes tested, it was observed that the sorption efficiency decreased with the increase in pH. In the article by T. Jóźwiak et al. [1], in which they investigated the effects of the degree of chitosan deacetylation on the sorption of RB5 dye from aqueous solutions, they obtained the highest sorption capacity at a pH of 4, while above this pH the sorption efficiency decreased for all tested sorbents. Moreover, in the article by U. Filipkowska et al. [35], in their study of the sorption efficiency of the dyes RB5 and RY84 using chitin and chitin subjected to the ammonification process as sorbent, they showed that the adsorption of both anionic dyes is most effective at low pH values. For chitin, the pH value is 2–4, while for modified chitin the sorption of dyes was highest at pH 2–3. At higher pH values, a decrease in efficiency was also observed as the pH of the solution increased.

### 2.3. Determination of the Sorption Equilibrium Time

**Figure 3:**
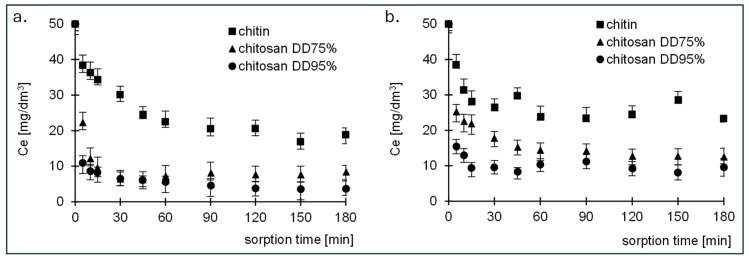
Changes in (**a**) RB5 and (**b**) RY84 dye concentration over time during adsorption onto chitin, chitosan DD75 and chitosan DD95%.

The effect of time on the removal efficiency of RB5 and RY84 dyes by adsorption on chitin, chitosan DD75 and chitosan DD95% was evaluated by the changes in the concentration of dye remaining in the solution after time t. Sorption was carried out at an optimum pH value, which was pH 3 for chitin and chitosan DD75 and pH = 4 for chitosan DD95%. The initial concentration of the dyes was 50 mg/dm^3^. The results are shown in Figure 3. 

The sorption equilibrium time for both dyes was longer for chitin and was 90 min. For the two tested chitosan samples, equilibrium was reached after a much shorter time of 30 min.

From the test results, it can be concluded that the change in the concentration remaining in the solution in case of chitin has three different zones: The first phase took place between 5–15 min, during which there was immediate adsorption of the dyes (the remaining concentration decreased by about 30%), indicating rapid external diffusion and adsorption on the surface of the adsorbent (Table 2). The second phase lasted between 20 and 85 min, during which the last free active centres were saturated by the dye molecules. After the second phase, the system entered a state of equilibrium.

The literature data confirm that the sorption equilibrium time depends on the type of adsorbent, the adsorbate and the process conditions [5,36,37]. In their study on the use of chitin and chitosan for the removal of the reactive dye Reactive Black 5, P. Szymczyk et al. [38] achieved the sorption equilibrium time after 360 min for chitin and 72 h for chitosan. G. Gibbs et al. [39] showed in their study of the sorption of the dye Acid Green 25 by chitosan that the adsorption process of the dye was completed after 1–2 h. On the other hand, K. Azlan et al. [40] showed in their studies on the sorption of acid dyes using chitosan and chemically modified chitosan as sorbents that the sorption equilibrium time for Acid Red 37 was 100 min.

### 2.4. Determination of the Kinetics of Dye Sorption onto Tested Sorbents

**Figure 4:**
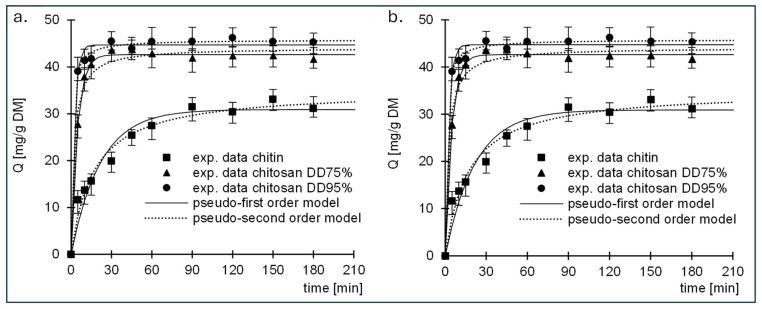
Sorption kinetics of (**a**) RB5 and (**b**) RY84 onto chitin, chitosan DD75 and chitosan DD95.

Experimental data from studies on the kinetics of sorption of RB5 and RY84 to chitin and chitosan (DD75% and DD95%) are described by pseudo-first-order and pseudo-second-order models (Figure 4, Table 1). In each research series, the pseudo-second order model showed better fit to the obtained data, which is a typical result for the sorption of organic dyes on biosorbents.

**Figure 5:**
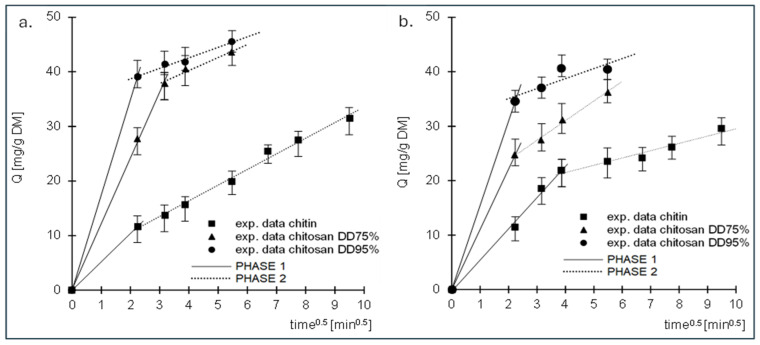
Intraparticle diffusion model of the sorption of (**a**) RB5 and (**b**) RY84 onto chitin, chitosan DD75 and chitosan DD95.

The data are also described by the intramolecular diffusion model (Figure 5, Table 2). Analysis of the data presented in the graphs shows that sorption of RB5 and RY84 occurred in 2 phases for each tested sorbent.

The first phase of sorption was characterised by high intensity but short duration. The dye ions diffused from the solution into the vicinity of the sorbent and then attached to the sorption centres on its surface. When the dye ions had saturated most of the active sites on the surface of the sorbent, the second phase began. Phase two was characterised by a longer duration but lower intensity than phase one. In this phase, the dye ions were bound to the less accessible sorption centres in the deeper layers of the sorbent. Due to the poorer availability of active sites, this phase was characterised by a much lower intensity than the first phase and a longer duration. After the last free active sites available in the sorption structure were saturated, the system reached equilibrium.

The values of qe, (cal.) determined by the pseudo-second-order model and the values of k_d1_ determined by the intramolecular diffusion model show that the sorption of RB5 and RY84 on the tested sorbents increased in the following order: chitin < chitosan DD75% < chitosan DD95%. The result is due to the increasing number of primary amino groups in this series, which are the most important sorption centres for anionic dyes.

### 2.5. Determination of the Sorption Capacity of Individual Dyes

The sorption capacity of the tested sorbents was determined at initial concentrations of the colourants RB5 and RY84 of 5–500 mg/dm^3^ (chitin) and 5–1000 mg/dm^3^ (chitosan DD75% and DD95%). This study was conducted at a pH and equilibrium time determined in an earlier phase.

**Figure 6:**
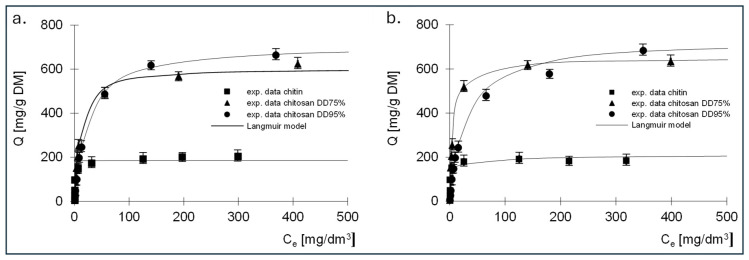
Isotherms of (**a**) RB5 and (**b**) RY84 sorption onto chitin, chitosan DD75 and chitosan DD90 (sorbent dose 1 g/dm^3^). Langmuir model.

**Figure 7:**
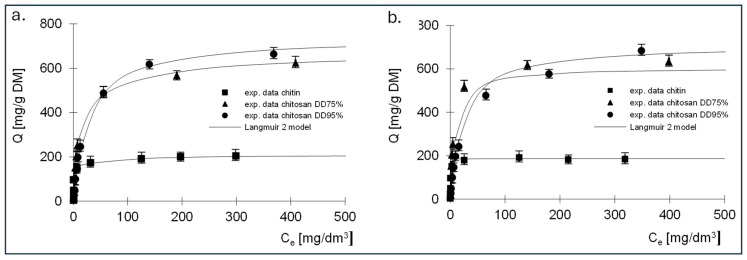
Isotherms of (**a**) RB5 and (**b**) RY84 sorption onto chitin, chitosan DD75 and chitosan DD90 (sorbent dose 1 g/dm^3^). Langmuir 2 model.

**Figure 8:**
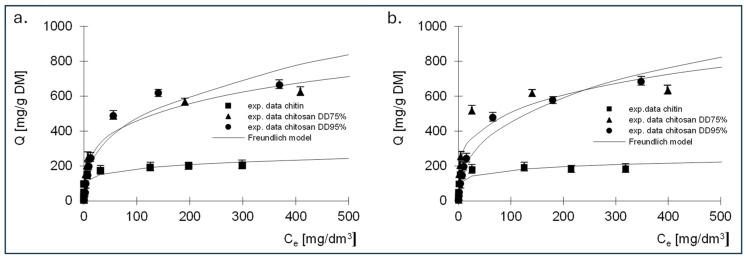
Isotherms of (**a**) RB5 and (**b**) RY84 sorption onto chitin, chitosan DD75 and chitosan DD90 (sorbent dose 1 g/dm^3^). Freundlich model.

The experimental results showing the relationship between the amount of adsorbed dye and the equilibrium concentration, as well as the Langmuir, Langmuir 2 and Freundlich isotherms determined on their basis, are shown in Figure 6, Figure 7 and Figure 8. The constants determined from Equations (5)–(7) for all dyes tested are listed in Table 3, Table 4, Table 5, Table 6, Table 7 and Table 8.

The best fit of the experimental data was obtained for the Langmuir 2 model; therefore, the mechanism of dye sorption was discussed based on this model. The presented data show that, regardless of the dye tested, the adsorption capacity of chitin at 211 mg/g DM for RB5 and 192 mg/g DM for RY84 was three times lower compared to chitosan adsorbents (680–742 mg/g DM for RB5 and 650–760 mg/g DM for RY84).

Analysis of the results of the constants from Langmuir’s Equation (2), shown in Table 5 and Table 6, indicates that there are two types of sorption centres in the tested sorbents.

A different mechanism of dye binding on the three tested adsorbents is evidenced by the values of the *K* constants describing the affinity of the adsorbate to the adsorbent (Table 4 and Table 6). The *K*_1_ constants determined for the active sites of the first type for chitin were high for both dyes and amounted to 6.28 and 3.92 dm^3^/mg, which could indicate strong binding of the dyes to chitin. The values of the *K*_1_ constants for chitosan DD75%, determined using the Langmuir double model, were an order of magnitude lower, ranging from 0.186 dm^3^/mg for RB5 to 0.23 dm^3^/mg for RY84. In case of the third sorbent with the highest DD95%, a further decrease in *K*_1_ was observed, amounting to 0.022 dm^3^/mg and 0.13 dm^3^/mg for RB5 and RY84, respectively.

The values of the *K*_2_ constants that describe the affinity for type II active sites were lower than the values of *K*_1_ regardless of the type of adsorbent and ranged from 0.017 (RY84, chitosan DD95%) to 0.581 dm^3^/mg (RY84, chitin). It should be noted that for both dyes, an increase in the degree of deacetylation of the sorbent led to a decrease in the solids content of *K*_1_ and *K*_2_. This could indicate that the binding to the sorbent with a higher degree of deacetylation is of a different nature than the binding to chitin.

The high affinity of the dye for chitin is due to the ability to bind the tested dyes on a positively charged surface through both electrostatic interactions and hydrogen bonding. This could indicate a stronger binding energy of the dye with chitin and a more chemical nature of the bond. Lower affinity values could indicate a physical bond.

This is confirmed by the sorption capacities determined for two types of active sites. In case of chitin, the *b*_1_ values describing the capacities at the active sites of the first type were about three times higher than the *b*_2_ values describing the capacities at the active sites of the second type. In case of chitosan DD95%, an inverse relationship was observed. The *K*_2_ values were higher than the *K*_1_ values and the *b*_2_ values were also higher than the *b*_1_ values.

The sorption capacities achieved in this study were comparable to or higher than those achieved by other researchers. The amount of bound dye depended on the way the chitin was prepared—flakes, hydrogel beads—and on the type of dye. In their study, Kurnia et al. obtained chitin capacities ranging from 38.21 to 45.87 mg/g [41].

The adsorption and desorption of malachite green with chitosan beads in deep eutectic solvents presented in the work of Sadiq et al. resulted in a capacity of 4.4 mg/g [42]. Tang et al., who investigated the sorption of malachite green, determined an adoption capacity of 29.5 mg/g for chitin hydrogels [43]. The capacities obtained in this study were much higher and amounted to 211 mg/g DM (RB5) and 192 mg/g DM (RY84) for chitin. The removal efficiencies of the two tested dyes on chitosan DD75% and DD95% were also high and were 680 and 650 mg/g DM for chitosan DD75% and 742 and 760 mg/g DM for chitosan DD95% for RB5 and RY84, respectively.

### 2.6. Determination of the Sorption Capacity of Dyes from Mixtures

In dyeing wastewater, dyes are usually found in two- and multi-component mixtures. Compared to single dyes, the adsorption of mixtures can be complex, both because of the potential for interactions between the dyes in solution and their ability to compete for active sites on the surface of the adsorbent. In addition, the adsorption of each individual dye may alter the surface charge of the adsorbent and consequently cause a decrease or increase in the binding efficiency of the other dyes present in the mixture. The aim of the study was to investigate the possibility of using chitosan sorbents for the sorption of RB5 and RY84 dyes from their mixture of aqueous solutions.

The studies were carried out in two variants—with a constant RY84 concentration of 50 mg/dm^3^ and a variable RB5 concentration of 5 to 500 mg/dm^3^ for chitin and 5 to 1000 mg/dm^3^ for chitosan DD75% and DD95%, and with a constant RB5 concentration of 50 mg/dm^3^ and a variable RY84 concentration of 5 to 500 mg/dm^3^ for chitin and 5 to 1000 mg/dm^3^ for chitosan DD75% and DD95%.

**Figure 9:**
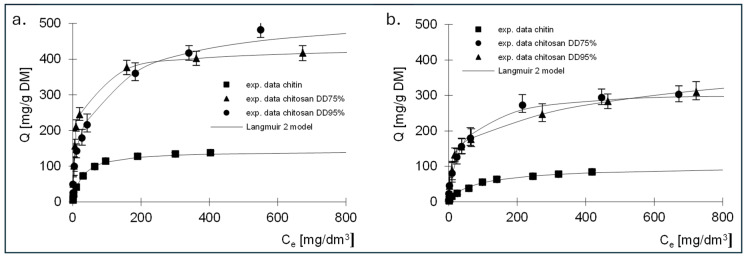
Isotherms of sorption of (**a**) RB5 and (**b**) RY84 from mixture dyes onto a. chitin, b. chitosan DD75% and c. chitosan DD90 (sorbent doze 1 g/dm^3^) (a. const. conc. RY84–50 mg/dm^3^, b. const. conc. RY84–50 mg/dm^3^).

The results of the studies on the sorption of the dye with variable concentration and constants determined using the Langmuir 2 model are shown in Figure 9 and in Table 9 and Table 10. 

Based on the obtained results, it can be seen that the sorption mechanism of the two tested dyes has changed. A lower sorption capacity was observed for the three sorbents. A greater decrease in the amount of dye absorbed was observed for RY84, which was 47.7% for chitin, 51% for chitosan DD75% and 42.5% for chitosan DD95%. In case of RB5, the effect of the presence of a second dye was lower and resulted in a decrease in the amount of bound dye by 32% for chitin, 36% for chitosan DD95% and 28.8% for chitosan DD95%.

A decrease in affinity was observed on the first type sites for chitin and chitosan DD75% (constant *K*_1_–Table 6). This trend was particularly visible for chitin, where the *K*_1_ value decreased from 6.28 to 0.031 dm^3^/mg for RB5 and from 3.92 to 0.009 dm^3^/mg for RY84. This could indicate that the presence of a second dye changed the nature of the binding from chemical to physical. No such phenomenon was observed in case of chitosan DD95%. Already in the sorption of dyes from single solutions, the obtained values of *K*_1_ and *K*_2_ solids indicated a more physical nature of sorption compared to chitin and chitosan DD95%.

In addition, the sorption capacities at the active sites of the second type were higher than at the sites of the first type, just as in the sorption of dyes from simple solutions.

Thus, increasing the degree of deacetylation of chitosan not only increased the overall sorption capacity, but also the binding method of the dyes tested.

**Figure 10:**
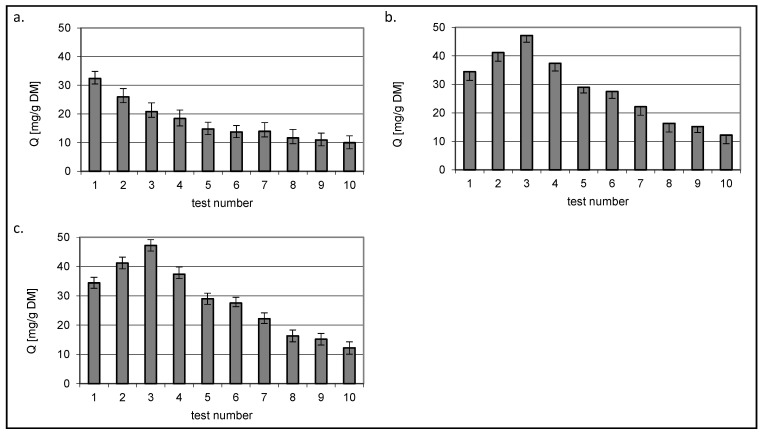
Isotherms of RB5 sorption from mixture dyes onto (**a**) chitin, (**b**) chitosan DD75% and (**c**) chitosan DD95% at constant RB5 concentration 50 mg/dm^3^ (sorbent dose 1 g/dm^3^).

**Figure 11:**
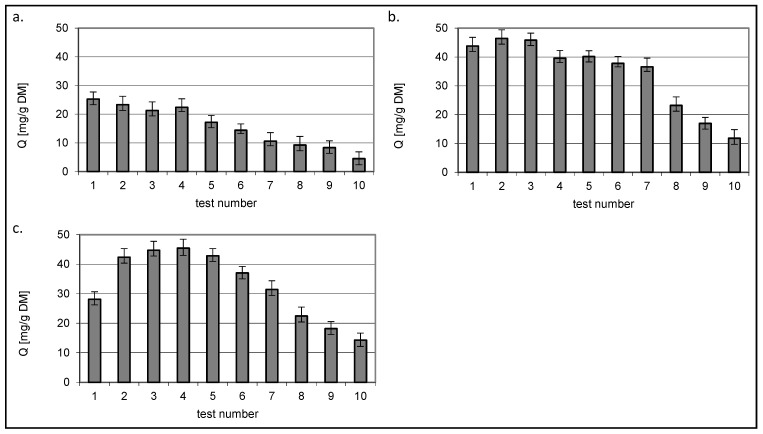
Isotherms of RY84 sorption from mixture dyes onto (**a**) chitin, (**b**) chitosan DD75% and (**c**) chitosan DD95% at constant RY84 concentration 50 mg/dm^3^ (sorbent dose 1 g/dm^3^).

Figure 10 and Figure 11 show the amount of dye removed that was present in the mixtures at a constant initial concentration of 50 mg/dm^3^. The test number indicates the consecutive concentration for which the values in Figure 8 and Figure 9 were determined.

Regardless of the sorbed colourant, a decrease in the Q value was observed for chitin with increasing concentration of the second dye present in the mixture.

A different relationship was observed for the two chitin sorbents DD75% and DD95%. The amount of bound dye in constant concentration mixtures reached the highest value in the third test where the variable concentration dye was present at a concentration of 50 mg/dm^3^, i.e., when the concentrations of both dyes were equal.

In the dyeing processes in the industries that use dyes, especially in the textile industry, a variety of dyes are used, which can be found in wastewater in the form of mixtures. The combination of different dyes in a mixture can affect the removal efficiency and make the removal process a challenge. The main reason for this is the presence of different functional groups in different dye classes. The amount of dye removed is also influenced by other factors such as pH or equilibrium time. Therefore, all parameters should be considered in the removal process. In the literature, studies on the removal of dye mixtures are not as commonly described as studies on the removal of dyes from single solutions. This may be caused by much more complicated and time-consuming research, but also by the need to describe the obtained results with a suitable model.

In this study, the double Langmuir model was used to describe the sorption of RB5 and RY84. The R^2^ coefficient values showed a very good fit of the Langmuir 2 isotherm, both in the case of sorption of dyes from single solutions and from their mixtures. Yu et al. [44] also showed that the adsorption amount and the equilibrium amount of the single dye system and the mixed system were consistent with the Langmuir model and the extended Langmuir isotherm.

Studies by Mavinkattimath et al. [45], Regti [22] and Giwa et al. [46] have also shown that the pH at which the sorption process is most effective depends on the type of sorbent and dye but is analogous for individual dyes and their mixtures. The same results were obtained in this study. This is very important when planning the use of sorption for the removal of dyes under real conditions, where we are mostly dealing with dye mixtures.

The adsorption capacity when sorbed from solutions of single dyes is always higher than the amount of removed dye when sorbed from the mixture. The reason for this is the competition of the dyes present in the mixture for the active sites of the sorbent. However, the total amount of dye removed is comparable to or higher than when sorbed from single solutions. The competition is also due to the fact that the tests were performed for dyes of the same type—cationic or anionic. This is justified because in practice one type of dye is used for colouring during a process.

Şahin [47] investigated the sorption of mixtures of reactive dyes on wool, Regti et al. [22] investigated the sorption of basic dyes on two types of activated carbon, Giwa et al. [46] researched competitive biosorptive removal of a basic dye from a ternary dye mixture using sawdust, and Mavinkattimath et al. [45] explored the simultaneous adsorption of Remazol brilliant blue and Disperse orange dyes on red mud.

## 3. Materials

### 3.1. Dyes

The dyes used in the study, Reactive Black 5 (RB5) and Reactive Yellow 84 (RY84), are anionic dyes produced by the dye factory ZPB “Boruta” SA in Zgierz (Table 11).

### 3.2. Sorbents

Chitin in the form of flakes with a degree of deacetylation DD = 35% and chitosan (DD 75 and DD95) from Heppe Medical Chitosan GmbH in Halle were used for this study. The source of the raw material was crab shells. Chitosan was characterised by a viscosity of 100 mPas, ash content ≤ 1%, a dry matter content > 85% and a heavy metal content Hg ≤ 0.2 ppm and Cd- ≤ 0.5 ppm. 

## 4. Methodology

### 4.1. Investigation of the Influence of the pH Value on the Efficiency of the Sorption of Dyes

This study began with the preparation of RB5 and RY84 working solutions at a concentration of 50 mg/dm^3^. An amount of 25 mg of each dye was weighed into beakers with a capacity of 1000 cm^3^ and then 500 cm^3^ of water with an appropriate pH was added. The pH ranged from 2 to pH 11. The 0.1 and 0.01 M NaOH (pH increase) or 0.1 and 0.01 M HCl (pH decrease) solutions were used to correct the pH.

Quantities of 0.1 g of chitin, chitosan DD75% and chitosan DD95% and 100 cm^3^ of the working solution of the tested dye with a pH of 2–11 were weighed into conical Erlenmeyer flasks with a capacity of 250 cm^3^. The flasks were then placed on a multistage magnetic stirrer MS-53M (JEIO TECH, Daejeon, Republic of Korea) with a mixing speed of 220 rpm. The concentration of the dye in the solution before and after sorption was measured using the spectrophotometric method. The UV-3100PC spectrophotometer (VWR spectrophotometers, VWR Interna-tional LLC., Mississauga, ON, Canada) was used to measure the absorbance of all samples at a wavelength of 600 nm for RB5 and 361 nm for RY84. Table 12 shows the parameters of the studies to determine the influence of pH on the sorption efficiency of the dyes.

### 4.2. Studies on the Influence of Time on the Effectiveness of Dyes

Six beakers with a capacity of 1000 cm^3^ were weighed with 50 mg of a given dye, then 1000 cm^3^ of water with the optimum pH determined for each dye and sorbent in the previous phase of this study and 1 g of sorbent were added. Table 13 shows the parameters of the tests to determine the sorption equilibrium time for the sorbents and dyes tested.

### 4.3. Testing the Efficiency of Sorption Capacity against Single Dyes

This study began with the preparation of a stock solution. For this purpose, 1 g of the dyes RB5 and RY84 were weighed out in powder form. Each dye was then quantitatively transferred to 1000 cm^3^ volumetric flasks to which distilled water was added at a pH determined for each dye and sorbent. The concentration of the dye in the solution was 1000 mg/dm^3^. Working solutions with an optimum pH value were then prepared from the stock solutions in PP containers (100 cm^3^). The concentrations of the dyes in the working solutions ranged from 5 to 500 mg/dm^3^ for chitin and from 5 to 1000 mg/dm^3^ for chitosan DD75% and chitosan DD95%. Before starting sorption, the initial concentration of each sample was measured with a spectrophotometer at a wavelength of 600 nm for RB5 and 361 nm for RY84.

Then, 0.1 g of chitin, chitosan DD75% and chitosan DD95% were weighed into 250 cm^3^ conical Erlenmeyer flasks and the prepared dye solutions were added at the appropriate concentrations. The flasks were placed on a magnetic stirrer for a specific sorption equilibrium time. At the end of the sorption process, the concentration of dyes remaining in the solution was determined. Table 14 shows the parameters of the tests to determine the sorption capacity of the sorbents tested.

### 4.4. Testing the Sorption Capacity of Dye Mixtures

The next phase of this study began with the preparation of basic dye solutions with a concentration of 2000 mg/dm^3^. For this purpose, 2 g each of the dyes RB5 and RY84 were weighed on an analytical balance, quantitatively transferred to volumetric flasks with a capacity of 1000 cm^3^, and water with the corresponding pH value was added.

Then, 50 cm^3^ of the working solution with concentrations from 10 to 1000 mg/dm^3^ (chitin) and from 10 to 2000 mg/dm^3^ (chitosan DD75% and DD95%) were placed in PP containers. Then, 50 and 100 cm^3^ of the stock solutions were measured into 1000 cm^3^ beakers, andwater with the appropriate pH was added. The next step was to add the solutions of a particular dye at a constant concentration to the solutions prepared in 50 cm^3^ PP containers. After mixing the solutions of the two dyes, the initial concentration of the dye mixture was read at wavelengths of 600 nm and 361 nm. For each combination of dye concentrations, a conversion factor was determined to convert the absorbance reading to the value of the concentration remaining after sorption. Table 15 shows the parameters of the tests to determine the sorption capacity in relation to the dye mixture.

The 0.1 g of chitin, chitosan DD75% and DD95% were weighed into 250 cm^3^ Erlenmeyer flasks and 100 cm^3^ of prepared solutions of a dye mixture with variable concentration of one of the dyes was added. The samples were placed on a magnetic stirrer. After the sorption process, samples were taken to measure the concentration of dye remaining in the solution. The concentrations were read at two wavelengths using a spectrophotometer.

### 4.5. FTIR Analysis

FTIR analysis of the sorbents tested was performed using the FT/IR-4700LE spectrometer with a single reflective diamond crystal ATR attachment (JASCO International, Tokyo, Japan). The scanning range of the samples ranged from 4000 to 400 cm^−1^. The resolution of the individual spectra was 1 cm^−1^. A total of 64 spectra were recorded for each sample and the results were then averaged. Before each measurement, the diamond crystal in the ATR was thoroughly cleaned with acetone and dried with a paper towel, after which a baseline correction was performed.

### 4.6. Calculation Methods

The amount of adsorbed dye was calculated from the relation (1):

(1) $$Q_{s}=\frac{\left(C_{o}-C_{s}\right)}{m}$$

Q_S_—mass of adsorbed dye [mg/dm^3^];

C_O_—initial concentration of dye [mg/dm^3^];

C_S_—concentration of dye after sorption [mg/dm^3^];

m—mass of sorbent [g DM].

The kinetics of dye sorption onto tested sorbents was described using pseudo-first-order (2), pseudo-second-order (3) and intraparticle diffusion (4) models:

(2) $$q=q_{e}\cdot \left(1-e^{\left(-k_{1}\cdot t\right)}\right)$$

(3) $$q=\frac{\left(k_{2}\cdot {q_{e}}^{2}\cdot t\right)}{\left(1+k_{2}\cdot q_{e}\cdot t\right)}$$

(4) $$q=k_{id}\cdot t^{0.5}$$

q—instantaneous value of sorbed dye [mg/g];

q_e_—the amount of dye sorbed at the equilibrium state [mg/g];

t—time of sorption [min];

k_1_—pseudo-first order adsorption rate constant [1/min];

k_2_—pseudo-second order adsorption rate constant [g/(mg × min)];

k_id_—intraparticular diffusion model adsorption rate constant [mg/(g × min^0.5^)].

The equation was used to determine the maximum adsorption capacity of the tested sorbents:

Langmuir’s isotherm (5):

(5) $$Q_{e}=\frac{b\cdot K\cdot C_{e}}{1+K\cdot C_{e}}$$

Q_e_—equilibrium amount of sorbed dye [mg/g DM];

b—maximum sorption capacity [mg/g DM];

K—constant used in the Langmuir equation [dm^3^/mg];

C_e_—concentration of dye remaining in the solution [mg/dm^3^].

Double Langmuir isotherm (6):

(6) $$Q_{e}=\frac{b_{1}\cdot K_{1}\cdot C}{1+K_{1}\cdot C}+\frac{b_{2}\cdot K_{2}\cdot C}{1+K_{2}\cdot C}$$

Q_e_—mass of sorbed dye [mg/g DM];

b_1_—maximum sorption capacity of the sorbent (type I active sites) [mg/g DM];

b_2_—maximum sorption capacity of the sorbent (type II active sites) [mg/g DM];

K_1_, K_2_—constants in Langmuir’s equation 2 [dm^3^/mg];

C_e_—concentration of dye remaining in the solution [mg/dm^3^].

Freundlich isotherm (7):

(7) $$Q_{e}=k\cdot C_{e}^{n}$$

Q_e_—actual sorption of sorbate on the sorbent [mg/g DM];

k—sorption equilibrium constant used in Freundlich’s model;

C_e_—concentration of dye remaining in the solution [mg/dm^3^];

n—heterogeneity parameter.

## 5. Conclusions

Studies on the sorption of dye mixtures, especially using chitosan sorbents, are of key importance due to the environmental impact of textile dyes in wastewater. Understanding the factors that influence sorption yield, such as the form and degree of deacetylation of chitosan, could lead to the development of more effective methods for removing dyes from aqueous solutions.

A research problem that should be considered at the same time is the fact that impurities such as dyes usually occur as mixtures. Studies on the sorption of dye mixtures are important for the development of models to predict rate constants, equilibrium time, sorption rate or sorption capacity based on factors such as pH, sorbent dose or dye concentration.

In this study, the efficiency of anionic adsorption of the dyes Reactive Black 5 and Reactive Yellow 84 and their mixtures from aqueous solutions was investigated. The sorbents used in this study were chitin, chitosan DD75% and chitosan DD95%.

– The efficiency of sorption of dyes onto chitosan sorbents depends on the pH value of the solution. The adsorption of the dyes RB5 and RY84 was most effective at a pH value of 3 for chitin and chitosan DD75% and at a pH value of 4 for chitosan DD95%.
– The investigations carried out allow for the conclusion that the chitosan sorbents used are efficient biosorbents regarding anionic dyes.
– The chitosan sorbents used in this study showed comparable sorption efficiency for both dyes, both in the sorption of single dyes and in the sorption of mixtures. The sorption efficiency depended much more on the type of sorbent than on the type of dye.
– The use of the Langmuir 2 model to describe the maximum sorption capacity results indicates the presence of two types of active sites in the structure of the sorbents used.
– The maximum sorption capacity of the used sorbents increased with increasing deacetylation rate in case of chitin to 211 and 192 mg/g DM for RB5 and RY84, respectively, for chitosan DD75% to 680 and 650 mg/g DM for RB5 and RY84, and for chitosan DD95% to 742 and 760 mg/d DM for RB5 and RY84, respectively.
– The sorption studies of the dye mixture indicate that the tested dyes, RB5 and RY84, compete for the active sites of the chitosan sorbents. The sorption capacity determined for the dyes during sorption and mixing was lower than for sorption from single solutions.

## Figures and Tables

**Table 1:** Kinetic parameters of dye sorption onto chitin, chitosan DD75% and chitosan DD95%.

Sorbent	Dye	Conc.	Pseudo-First-Order Model			Pseudo-Second-Order Model			Exp. Data	Equil. Time
			k_1_	q_e, (cal.)_	R^2^	k_2_	q_e, (cal.)_	R^2^		
		[mg/dm^3^]	[1/min]	[mg/g]	-	[g/mg × min]	[mg/g]	-	[mg/g]	[min]
Chitin	RB5	50	0.0460	30.91	0.9511	0.0017	35.08	0.9752	31.47	90
	RY84	50	0.1043	27.36	0.9645	0.0048	29.90	0.9846	29.56	90
Chitosan DD75	RB5	50	0.2122	42.62	0.9795	0.0103	44.14	0.9814	43.56	30
	RY84	50	0.1803	36.02	0.9743	0.0089	37.79	0.9927	36.21	30
Chitosan DD95	RB5	50	0.3855	44.72	0.9890	0.0229	45.82	0.9971	45.52	30
	RY84	50	0.3659	40.36	0.9911	0.0272	41.16	0.9921	40.43	30

**Table 2:** Dye diffusion rate constants determined using the intraparticle diffusion model.

Sorbent	Dye	Conc.	PHASE 1			PHASE 2		
			k_d1_	Time	R^2^	k_d2_	Time	R^2^
		[mg/dm^3^]	[mg/(g × min^0.5^)]	[min]	-	[mg/(g × min^0.5^)]	[min]	-
Chitin	RB5	50	5.201	5	0.(999)	2.869	85	0.9911
	RY84	50	5.731	15	0.9939	1.338	75	0.9476
Chitosan DD75	RB5	50	12.054	10	0.9993	2.369	20	0.9715
	RY84	50	11.055	5	0.(999)	3.617	25	0.9911
Chitosan DD95	RB5	50	17.477	5	0.(999)	1.926	25	0.9762
	RY84	50	15.465	5	0.(999)	1.834	25	0.8498

**Table 3:** Constants determined from the Langmuir model for RB5—sorption of single dyes.

Model	Constants in the Sorption Model	Sorbent		
		Chitin	Chitosan DD75%	Chitosan DD95%
Langmuir isotherm	*b*_1_ [mg/g DM]	186.60	606.13	710.81
	*K*_1_ [dm^3^/mg]	3.68	0.102	0.042
	R^2^ [-]	0.972	0.988	0.999

**Table 4:** Constants determined from the Langmuir model for RY84—sorption of single dyes.

Model	Constants in the Sorption Model	Sorbent		
		Chitin	Chitosan DD75%	Chitosan DD95%
Langmuir isotherm	*b*_1_ [mg/g DM]	182.60	638.81	698.92
	*K*_1_ [dm^3^/mg]	3.18	0.176	0.037
	R^2^ [-]	0.994	0.997	0.994

**Table 5:** Constants determined from the Langmuir 2 model for RB5—sorption of single dyes.

Model	Constants in the Sorption Model	Sorbent		
		Chitin	Chitosan DD75%	Chitosan DD95%
Langmuir 2 isotherm	*Q*_max_ [mg/g DM]	211	680	742
	*b*_1_ [mg/g DM]	150	426	304
	*K*_1_ [dm^3^/mg]	6.28	0.186	0.022
	*b*_2_ [mg/g DM]	61	254	438
	*K*_2_ [dm^3^/mg]	0.0162	0.010	0.047
	R^2^ [-]	0.996	0.994	0.997

**Table 6:** Constants determined from the Langmuir 2 model for RY84—sorption of single dyes.

Model	Constants in the Sorption Model	Sorbent		
		Chitin	Chitosan DD75%	Chitosan DD95%
Langmuir 2 isotherm	*Q*_max_ [mg/g DM]	192	650	760
	*b*_1_ [mg/g DM]	151	510	211
	*K*_1_ [dm^3^/mg]	3.92	0.23	0.13
	*b*_2_ [mg/g DM]	40	140	529
	*K*_2_ [dm^3^/mg]	0.581	0.053	0.014
	R^2^ [-]	0.992	0.998	0.998

**Table 7:** Constants determined from the Freundlich model for RB5—sorption of single dyes.

Model	Constants in the Sorption Model	Sorbent		
		Chitin	Chitosan DD75%	Chitosan DD95%
Freundlich isotherm	*k*	83.95	131.55	89.53
	*n*	0.171	0.272	0.36
	R^2^ [-]	0.914	0.962	0.925

**Table 8:** Constants determined from the Langmuir 2 model for RY84—sorption of single dyes.

Model	Constants in the Sorption Model	Sorbent		
		Chitin	Chitosan DD75%	Chitosan DD95%
Freundlich isotherm	*k*	86.18	157.29	78.62
	*n*	0.155	0.255	0.380
	R^2^ [-]	0.844	0.898	0.966

**Table 9:** Constants determined from Langmuir 2 model for RB5 dye—sorption of dye mixtures (constant concentration RY84–50 mg/dm^3^).

Model	Constants in the Sorption Model	Sorbent		
		Chitin	Chitosan DD75%	Chitosan DD95%
**Langmuir 2 isotherm**	*Q*_max_ [mg/g DM]	143	435	528
	*b*_1_ [mg/g DM]	111	309	143
	*K*_1_ [dm^3^/mg]	0.031	0.126	0.240
	*b*_2_ [mg/g DM]	32	126	385
	*K*_2_ [dm^3^/mg]	0.083	0.010	0.003
	R^2^ [-]	0.992	0.994	0.991

**Table 10:** Constants determined from Langmuir 2 model for RT84 dye—sorption of dye mixtures (constant concentration RB5–50 mg/dm^3^).

Model	Constants in the Sorption Model	Sorbent		
		Chitin	Chitosan DD75%	Chitosan DD95%
**Langmuir 2 isotherm**	*Q*_max_ [mg/g DM]	100	237	437
	*b*_1_ [mg/g DM]	87	202	163
	*K*_1_ [dm^3^/mg]	0.009	0.010	0.200
	*b*_2_ [mg/g DM]	13	116	274
	*K*_2_ [dm^3^/mg]	0.37	0.053	0.014
	R^2^ [-]	0.992	0.991	0.991

**Table 11:** Characteristics of Reactive Black 5 and Reactive Yellow 84.

Name	Reactive Black 5–(RB5)	Reactive Yellow 84–(RY84)
Structural formula	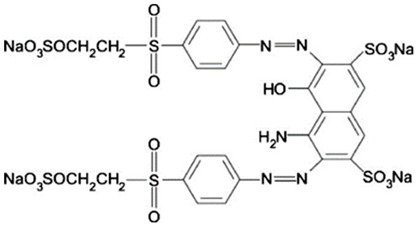	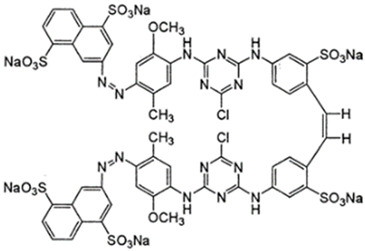
Molecular mass	991 g/mol	1701 g/mol
λmax	600 nm	361 nm
Character	Acidic (anionic–reactive)	Acidic (anionic–reactive)
Class	Diazo dye	Diazo dye
Type of active groups	Vinylsulfone	Chlorothriazine
Use	Dyeing cotton, viscose, wool, polyamide fibres	Dyeing polyester, cotton, rayon

**Table 12:** Parameters of this study to determine the effect of pH on the efficiency of sorption of dyes.

Type of Sorbent	Sorbed Dye	Dye Concentration [mg/dm^3^]	Initial pH of the Solution [pH]	Mixing Speed [r.p.m]	Sorption Time [min]	Temperature [°C]
Chitin	RB5/RY84	50	2–11	220	180	22
Chitosan DD75%						
Chitosan DD95%						

**Table 13:** Parameters of research on the determination of the sorption equilibrium time.

Type of Sorbent	Sorbed Dye	Dye Concentration [mg/dm^3^]	Reaction of the Dye Solution [Ph]	Mixing Speed [r.p.m]	Sorption Time [min]	Temp. [°C]
Chitin	RB5	50	3	220	0, 5, 10, 15, 30, 45, 60, 90, 120, 150, 180	22
	RY84					
Chitosan DD75%	RB5					
	RY84					
Chitosan DD95%	RB5		4			
	RY84					

**Table 14:** Parameters of research on the sorption capacity of sorbents.

Type of Sorbent	Sorbed Dye	Dye Concentration [mg/dm^3^]	Reaction of the Dye Solution [pH]	Mixing Speed [r.p.m]	Sorption Time [min]	Temp. [°C]
Chitin	RB5	5, 10, 25, 50, 100, 150, 200, 300, 400, 500	3	220	90	22
	RY84					
Chitosan DD75%	RB5	5, 25, 50, 100, 150, 200, 250, 500, 750, 1000			30	
	RY84					
Chitosan DD95%	RB5	5, 25, 50, 100, 150, 200, 250, 500, 750, 1000	4			
	RY84					

**Table 15:** Parameters of research on the sorption capacity of sorbents in relation to the mixture of dyes.

Type of Sorbent	Mixture	Dye	Dye Concentration [mg/dm^3^]	Reaction of the Dye Solution [pH]	Mixing Speed [r.p.m]	Sorption Time [min]	Temp. [°C]
Chitin	I	RB5	5, 10, 25, 50, 100, 150, 200, 300, 400, 500	3	220	90	22
		RY84	50				
	II	RB5	50				
		RY84	5, 10, 25, 50, 100, 150, 200, 300, 400, 500				
Chitosan DD75%	I	RB5	5, 25, 50, 100, 150, 200, 250, 500, 750, 1000	3		30	
		RY84	100				
	II	RB5	100				
		RY84	5, 25, 50, 100, 150, 200, 250, 500, 750, 1000				
Chitosan DD95%	I	RB5	5, 25, 50, 100, 150, 200, 250, 500, 750, 1000	4			
		RY84	50				
	II	RB5	50				
		RY84	5, 25, 50, 100, 150, 200, 250, 500, 750, 1000				

## Data Availability

The data presented in this study are available on request from the corresponding authors.
